# Development and Verification of Crack-Enriched Elements Based on XFEM

**DOI:** 10.3390/ma19061219

**Published:** 2026-03-19

**Authors:** Yanke Shi, Liming Chen, Pengtuan Zhao, Junyi Huo, Luyang Shi

**Affiliations:** School of Civil Engineering and Transportation, North China University of Water Resources and Electric Power, Zhengzhou 450045, China

**Keywords:** fracture failure, crack propagation, the extended finite element method, user-defined element, crack enrichment element

## Abstract

Concrete structures often develop penetrating cracks due to the initiation and propagation of local cracks during service, which may lead to the fracture and failure of the entire structure. The propagation modes and laws of cracks in structural members are closely related to the safety of the overall structure. Conducting research on crack propagation and predicting crack propagation paths for cracked structures can provide technical support for the safety design and reinforcement of structures. Based on the basic framework of the extended finite element method (XFEM), this paper develops a user-defined element (UEL) for ABAQUS using the level set method, and simulates in a two-dimensional space the crack propagation in concrete beam bending tests with the self-developed UEL and the built-in XFEM module of the software. The solution results of the self-developed UEL are consistent in trend with those of the XFEM module, yet the cracks simulated by the XFEM module can only propagate along element boundaries and cannot cross elements, and the accuracy of its results is highly dependent on mesh size. The crack tip simulated by the self-developed UEL can stay inside the element, and the simulated crack propagation paths show a higher degree of agreement with the experimental results. The correctness of the UEL is verified through comparative analysis with the results of the four-point bending tests of concrete beams and the XFEM module of the software. The UEL developed in this paper can effectively predict the crack propagation paths of concrete beams and reveal the multi-crack propagation laws of concrete beams.

## 1. Introduction

When structural members such as concrete beams serve under complex loads, natural defects including voids and microcracks inside them inevitably lead to the initiation of cracks in the structure. These initial cracks may propagate under external loading and, in severe cases, can cause fracture failure of concrete beams. The main approaches for investigating the crack propagation mechanism of concrete beams include theoretical analysis, experimental analysis, and numerical simulation. Numerous scholars have adopted various methods to study the cracking mechanism of concrete beams.

Traditional numerical methods such as the boundary element method, finite element method, and interface element method require remeshing when simulating crack propagation, and thus cannot effectively analyze crack propagation problems. In 1999, Belytschko et al. [1] and Moës et al. [2] from Northwestern University (USA) proposed a novel algorithm based on the finite element method, the extended finite element method (XFEM), which overcomes various drawbacks of the conventional finite element method in dealing with discontinuous media problems and lays a foundation for subsequent research on crack propagation.

The earliest study on structural fracture was conducted by Professor Griffith, who put forward the fracture energy balance theory that laid the foundation for fracture mechanics [3]. Orowan and Irwin [4] successively revised and developed this theory, which eventually led to the establishment of the discipline of Linear Elastic Fracture Mechanics (LEFM). When simulating crack propagation in quasi-brittle materials such as concrete, rock and masonry, the crack propagation paths are complex, which belongs to the discontinuous media problem in fracture mechanics [5]. Compared with other experimental methods, the finite element method (FEM) has become the most popular numerical method due to its simple implementation, wide applicability and low number of constraints [6], and has been widely used in the simulation and analysis of cracking processes in complex structures. When the finite element method is applied to discontinuous media problems, it requires that element boundaries coincide with geometric boundaries. This necessitates mesh refinement around defects such as voids and cracks, resulting in increased computational cost. Furthermore, remeshing around the crack tip is required as the crack propagates, and the crack can only extend along element boundaries, which leads to complicated programming and large-scale computation [7].

With the development of computer technology, a variety of numerical analysis software has emerged. The commercial finite element software ABAQUS is widely used owing to its convenient secondary development interface and unique advantages in solving nonlinear problems. Its built-in XFEM module can solve most cracking problems; however, certain simplifications have been adopted in the module integration to reduce solution difficulty, leading to some limitations and deficiencies [7]. Guan et al. [8] derived an analytical expression for the crack opening displacement of concrete bending beams during propagation based on the Paris displacement formula, and proposed a numerical method for simulating the whole process of crack initiation, propagation and instability failure of concrete cracks under loading. Hu [9] put forward a new method for calculating the initiation toughness and failure toughness of concrete beams. Hu [10] adopted a cohesive zone model to simulate the entire static fracture process of concrete beams under four-point bending, and verified the feasibility of the method by comparing numerical results with experimental data. Wu [11] analyzed the fracture process and influencing factors of crack propagation in concrete beams based on the standard XFEM in ABAQUS. The results show that, under a given load, the stress intensity factor of concrete increases with the increase in the initial crack/height ratio and decreases with the increase in the section height. Fang [12] also carried out crack propagation simulations on concrete beams using ABAQUS. Rui et al. [13] studied the stress intensity factors of K^I^, K^II^ and K^III^ results derived from ABAQUS^®^ under mixed-mode loading, and analyzed crack propagation and evolution through the thickness of the compact tension specimen. Justas [14] derived two universal computational models for flexural reinforced concrete (RC) members strengthened with externally bonded FRP (EB-FRP) and near-surface mounted FRP (NSM-FRP) reinforcements, by combining the principles of solid fracture mechanics and generally accepted fundamental assumptions. Xiao [15] studied the failure mode differences of UHPC and UHPFRC subjected to dynamic and quasi-static compressive loading. The results indicate that when using the XFEM module, the initial crack tips must be located at element boundaries in numerical simulation; otherwise, the bearing capacity of the member will be underestimated.

Based on the fundamental theory of the extended finite element method, this study develops an ABAQUS user-defined element (UEL) analysis program for XFEM using MATLAB R2025A and FORTRAN 2023. In crack propagation simulations, the program allows the crack tip to be positioned at an arbitrary location within an element, and enables the adjustment of enrichment functions at the crack tip and the domain of the J-integral, which can effectively reduce computational errors. Combined with the four-point bending test on concrete beams, crack propagation simulations are performed using both the built-in XFEM module of ABAQUS and the self-developed UEL program. By comparing the numerically obtained crack propagation paths with those measured in the experiment, it is demonstrated that the user-defined element program can effectively simulate crack propagation in concrete structures and reproduce the fracture process of concrete beams. This research can provide a certain reference basis for the construction and safety reinforcement of concrete beams.

## 2. Extended Finite Element Method for Cracked Concrete Bodies

### 2.1. Theory of the Level Set Method

The level set method (LSM) was first proposed by Osher and Sethian [16], which is a numerical technique for determining the interface position and tracking the interface movement. This method embeds the researched curve or surface into the level set function in a one-dimensional higher space $\Psi \left(X\left(t\right),t\right)$. Driven by a certain velocity field, the boundary motion analysis and tracking of the curve or surface are realized by solving the level set equation.

A moving interface $\gamma (t)\subset R^{2}$ can be expressed as the level set curve of the function $\Psi \left(X,t\right)$ in $R^{2}\times R\to R$, where

(1) $$\gamma (t)=\left\{X\in R^{2}:\Psi \left(X,t\right)=0\right\}$$

**Figure 1:**
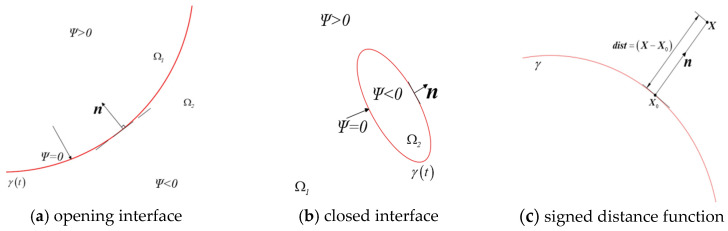
Schematic diagram of level set function.

The movement of $\gamma \left(t\right)$ can be derived from the evolution equation of Ψ [17]:

(2) $$\frac{\partial \Psi }{\partial t}+F|\nabla \Psi |=0,\;\;\Psi (X,0)\;\mathrm{is}\;\mathrm{given}$$

where *F* is the velocity of a point on the interface $X\in \gamma \left(t\right)$ along the outward normal direction of the interface (see Figure 1).

The initial condition $\Psi \left(X,0\right)$ is generally represented by a signed distance function, which takes a positive value on one side of the interface, a negative value on the other side, and a value of zero at the interface. To construct a level set function using the signed distance function, the nearest point $X_{0}$ to the observation point X on the interface is first determined, as shown in Figure 1c. The vector $dist=X-X_{0}$ is orthogonal to the interface at point $X_{0}$, and n is the unit normal vector of the interface at point $X_{0}$. The level set function is defined as

(3) $$\Psi (X,0)=(X-X_{0})\cdot n$$

**Figure 2:**
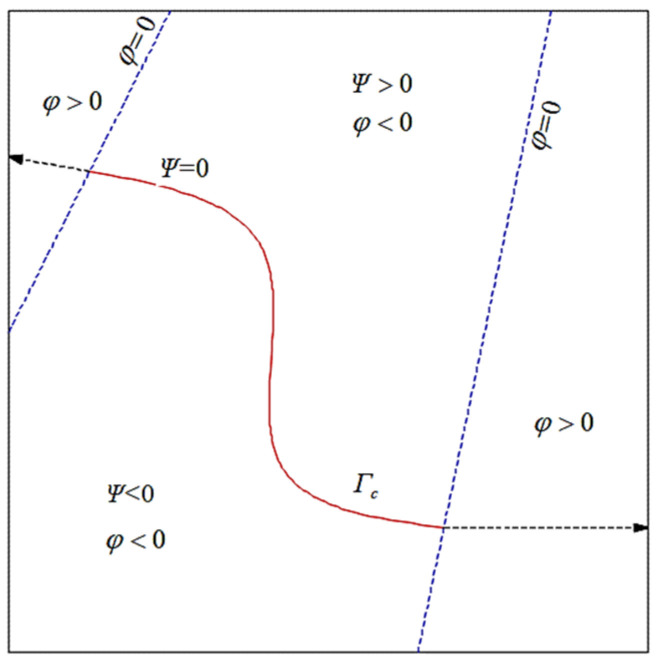
Level set function for internal cracks.

To construct the level set function over the entire domain, the two crack tips are extended tangentially along the directions of the crack tips to the domain boundary, as shown in Figure 2. The normal level set functions are calculated based on the actual crack segments $\Gamma_{c}$ and the virtual crack segments (the extended ones). The level set functions Ψ and φ can characterize the internal cracks of the structure: the positions where φ=0 and Ψ=0 correspond to the crack tips; the regions where Ψ=0 and φ≤0 represent the crack surfaces.

### 2.2. Displacement Mode

**Figure 3:**
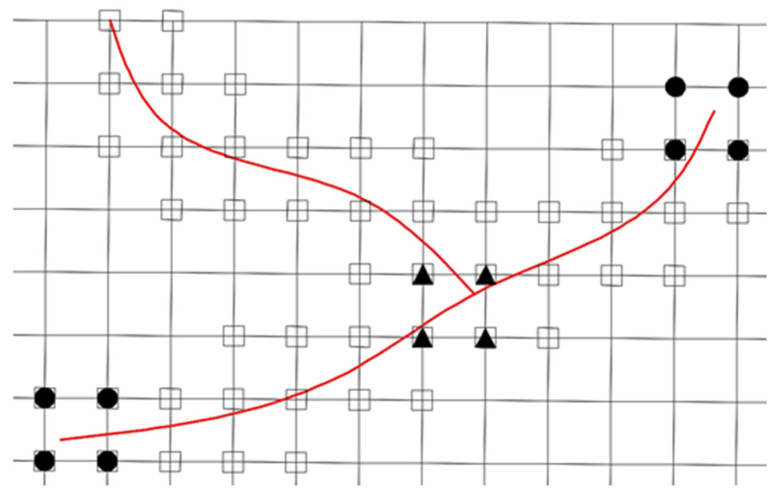
Enriched nodes and enrichment types.

The core idea of the Partition of Unity Method (PUM) is to partition the solution domain into subdomains to approximate local functions as accurately as possible, then “assemble” these subdomains to form a global approximation of the function. Based on the concept of the Partition of Unity Method, the structural body with multiple cracks is divided into conventional finite elements, through-crack elements, crack tip-containing elements and crack intersection elements. According to the **through-crack enrichment function** ***H***(***x***) and **crack tip enrichment function** ***F**_α_*(***x***), the intersection enrichment function is introduced to derive the XFEM displacement mode for multiple cracks. Figure 3 shows the finite element meshes of the structure with multiple cracks, and the mesh generation is independent of the crack positions.

The enriched displacement approximation using XFEM can be written as follows:

(4) $$u=\sum_{i\in N^{s}}N_{i}(x)u_{i}+\sum_{i\in N^{J}}\bar{N_{i}}(x)(H(x)-H(x_{i}))a_{i}+\sum_{i\in N^{K}}\bar{N_{i}}(x)\sum_{k}(F_{k}(x)-F_{k}(x_{i}))b_{i}^{k}+\sum_{q=1}^{nx}\sum_{i\in N^{M}}\bar{N_{i}}(x)J_{q}(x)c_{i}^{q}$$

where $N_{i}(x)$ is the finite element shape function; $u_{i}$, $a_{i}$, $b_{i}^{k}$ and $c_{i}^{q}$ are the displacements and enrichment nodal variables; I is the set of all nodes in the discretization; J is the set of nodes whose support is entirely split by the crack (the squared nodes in Figure 3) and are enriched with a modified Heaviside step function H(x), which assumes the value +1 above the crack and −1 below the crack; $K^{*}$ is the set of nodes whose support is partly split by the crack (the circled solid nodes in Figure 3); K is the set of nodes $K^{*}$ plus their neighboring nodes (the circled hollow nodes in Figure 3) and are enriched with the crack tip branch enrichment functions $F_{k}(x)$; M is the set of nodes whose support contains the junction and which are enriched with the junction enrichment function $J_{q}(x)$.

For isotropic elasticity material, the crack tip branch enrichment functions $F_{\alpha }(x)$ (α = 1, …, 4) are defined as [18]

(5) $$F_{\alpha }(x)=\left[\begin{array}{cccc} \sqrt{r}\sin\frac{\theta }{2} & \sqrt{r}\cos\frac{\theta }{2} & \sqrt{r}\sin\frac{\theta }{2}\sin\theta & \sqrt{r}\cos\frac{\theta }{2}\sin\theta \end{array}\right],\;\alpha =1,2,3,4$$

where r and θ are the local crack tip polar coordinates.

The junction enrichment function $J_{q}(x)$ is chosen as [19]

(6) $$\left\{\begin{array}{c} J_{q}\left(x\right)=H\left(f_{1}\left(x\right)\right)-H\left(f_{1}\left(x_{i}\right)\right)\;\;\;\;\;f_{1}\left(x\right)f_{1}\left(x_{i}\right)>0\\ J_{q}\left(x\right)=H\left(f_{2}\left(x\right)\right)-H\left(f_{2}\left(x_{i}\right)\right)\;\;\;\;\;f_{1}\left(x\right)f_{1}\left(x_{i}\right)<0 \end{array}\right.$$

where $f_{1}\left(x\right)$ and $f_{2}\left(x\right)$ are the signed distance functions of the master crack (the crack with two crack tips in Figure 3) and minor crack (the crack with one crack tip in Figure 3), respectively.

### 2.3. Governing Equations

After the construction of the displacement mode, the governing equations for the crack problem are derived by the principle of virtual work. The constitutive law in a linear elastic body on the conditions of small deformation is described as

(7) σ=C:ε

where C is the Hooke tensor, and the strain tensor ε can be expressed as

(8) $$\varepsilon =\frac{1}{2}\left[\nabla u+{\left(\nabla u\right)}^{T}\right]$$

where u can be obtained from Equation (4).

The weak form of the equilibrium equation is expressed:

For virtual displacement ∀δu∈V,

(9) $$\int_{\Omega }\sigma \left(u\right):\delta \varepsilon d\Omega +\int_{\Omega }b\cdot \delta ud\Omega =\int_{\Gamma_{t}}\bar{t}\cdot \delta ud\Omega$$

where σ is the Cauchy stress tensor, ε is the strain tensor.

The governing equation can be expressed:

(10) Kδ=f

where K and f are the global stiffness matrix and external nodal force vector, respectively; and f is related to the pressures on the structure.

The element contribution to K is as follows:

(11) $$k_{ij}^{e}=\left[\begin{array}{ccc} k_{ij}^{uu} & k_{ij}^{ua} & k_{ij}^{ub}\\ k_{ij}^{au} & k_{ij}^{aa} & k_{ij}^{ab}\\ k_{ij}^{bu} & k_{ij}^{ba} & k_{ij}^{bb} \end{array}\right]$$

where

(12) $$k_{ij}^{rs}={\int_{\Omega }\left(B_{i}^{r}\right)}^{T}DB_{j}^{s}d\Omega \;\;\;\;\;\;\;\;\left(r,s=u,a,b\right)$$

with

(13) $$B_{i}^{u}=\left[\begin{array}{cc} \frac{\partial N_{i}}{\partial x} & 0\\ 0 & \frac{\partial N_{i}}{\partial y}\\ \frac{\partial N_{i}}{\partial x} & \frac{\partial N_{i}}{\partial y} \end{array}\right]$$

(14) $$B_{i}^{a}=\left[\begin{array}{cc} \frac{\partial N_{i}(H-H(x_{i}))}{\partial x} & 0\\ 0 & \frac{\partial N_{i}(H-H(x_{i}))}{\partial y}\\ \frac{\partial N_{i}(H-H(x_{i}))}{\partial x} & \frac{\partial N_{i}(H-H(x_{i}))}{\partial y} \end{array}\right]=(H-H(x_{i}))\left[\begin{array}{cc} \frac{\partial N_{i}}{\partial x} & 0\\ 0 & \frac{\partial N_{i}}{\partial y}\\ \frac{\partial N_{i}}{\partial x} & \frac{\partial N_{i}}{\partial y} \end{array}\right]$$

(15) $$B_{i}^{b}=\left[\ldots \begin{array}{cc} \frac{\partial N_{i}(F_{k}-F_{k}(x_{i}))}{\partial x} & 0\\ 0 & \frac{\partial N_{i}(F_{k}-F_{k}(x_{i}))}{\partial y}\\ \frac{\partial N_{i}(F_{k}-F_{k}(x_{i}))}{\partial x} & \frac{\partial N_{i}(F_{k}-F_{k}(x_{i}))}{\partial y} \end{array}\ldots \right]\;\;(k=1,2,3,4)$$

Moreover, the element contribution to f is as follows:

(16) $$f_{i}^{e}=\left[f_{i}^{u}\;\;\;\;\;f_{i}^{a}\;\;\;\;f_{i}^{b}\right]$$

with

(17) $$f_{i}^{u}=\int_{\Gamma_{t}}N_{i}\bar{t}d\Gamma +\int_{\Omega }N_{i}bd\Omega$$

(18) $$f_{i}^{a}=\int_{\Gamma_{t}}N_{i}\left(H-H\left(x_{i}\right)\right)\bar{t}d\Gamma +\int_{\Omega }N_{i}\left(H-H\left(x_{i}\right)\right)bd\Omega \;\;$$

(19) $$f^{b}=\int_{\Gamma_{t}}N_{i}\left(\varphi_{\alpha }-\varphi_{\alpha }\left(x_{i}\right)\right)\bar{t}d\Gamma \;\;+\int_{\Omega }N_{i}\left(\varphi_{\alpha }-\varphi_{\alpha }\left(x_{i}\right)\right)bd\Omega \;\;\;(\alpha \;=\;1,\;\ldots ,\;4)$$

## 3. A User-Defined Element (UEL) for ABAQUS

To meet the users’ demand for developing custom elements to solve specific mechanical problems, the ABAQUS software platform provides a secondary development interface for the user-defined element (UEL) subroutine. In this study, an analysis program incorporating the 2D four-node crack enrichment element is developed through the integration of MATLAB, FORTRAN, ABAQUS 2024, and STUDIO 2024, which is used to address relevant engineering problems.

The crack enrichment element has 12 degrees of freedom (DOFs). In accordance with the DOF conventions of ABAQUS, DOFs 1 and 2 represent the displacements along the X and Y axes, respectively. DOFs 3 and 4 are additional DOFs associated with the Heaviside enrichment function. DOFs 5 to 7 and 11 to 15 denote additional DOFs related to the crack tip enrichment functions, while the unused DOFs are constrained.

The properties of the custom element are defined in the ABAQUS .inp file as follows:

*User Element, Nodes = 4, Type = U12, Properties = 2, Iproperties = 5, Coordinates = 2, VARIABLES = 1001

1, 2, 3, 4, 5, 6, 7, 11, 12, 13, 14, 15

*Element, type = U12, Elset = UEL

*Uel Property, Elset = UEL

**Figure 4:**
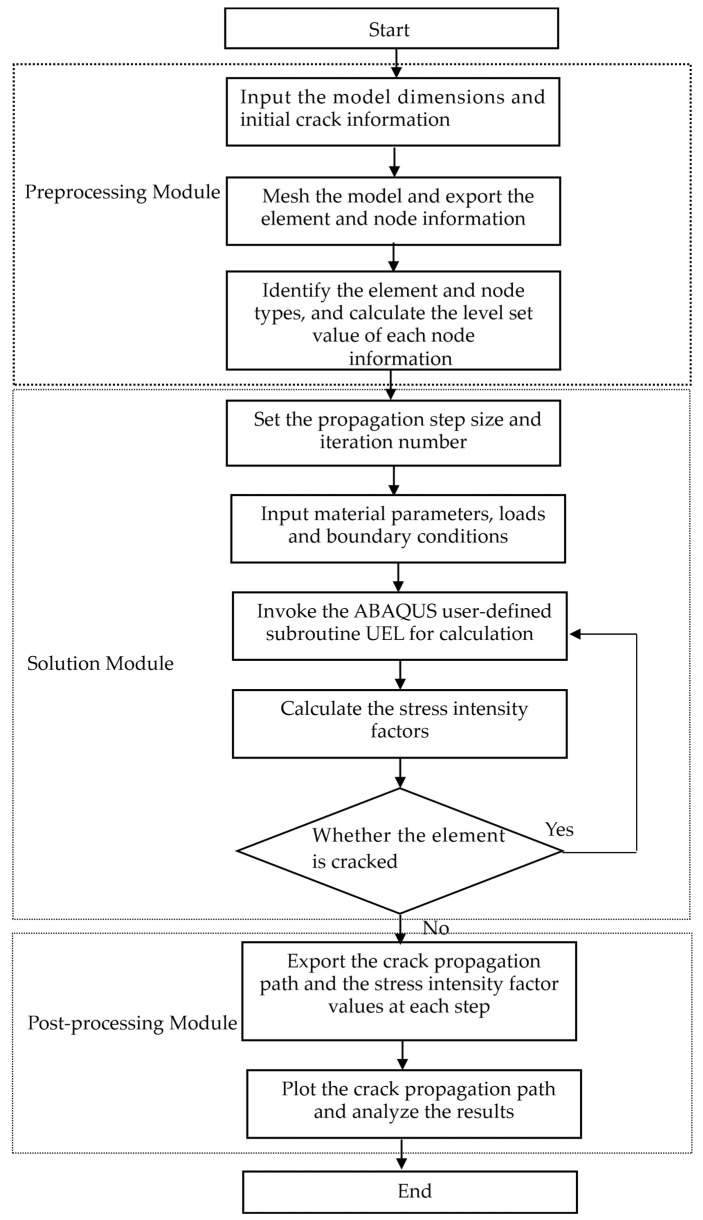
User-defined unit program flowchart.

The crack propagation simulation process in this paper is illustrated in Figure 4: First, ABAQUS, Visual Studio, and Fortran are associated and launched to prepare for calling the user-defined element (UEL) subroutine. Next, boundary conditions and load conditions are applied in the .inp file, and the corresponding material parameters are assigned. Then, the crack propagation step size and the number of propagation steps are set, and the MATLAB subroutine AutoABAQUS.m of the ABAQUS solving module is automatically called to perform the crack propagation simulation. After the crack propagation terminates, the cracking direction, crack tip coordinates, and stress intensity factors (SIFs) of each propagation step are stored in the result file in the form of a matrix. Finally, the data are processed using plotting software such as Origin or MATLAB, and the analytical solutions are compared with the calculation results of the custom element subroutine to analyze the influences of factors such as crack size, mesh density, J-integral enhancement range, and the number of crack tip enrichment element layers on the calculation results. The code can be seen in Appendix A.

## 4. Numerical Verification

**Figure 5:**
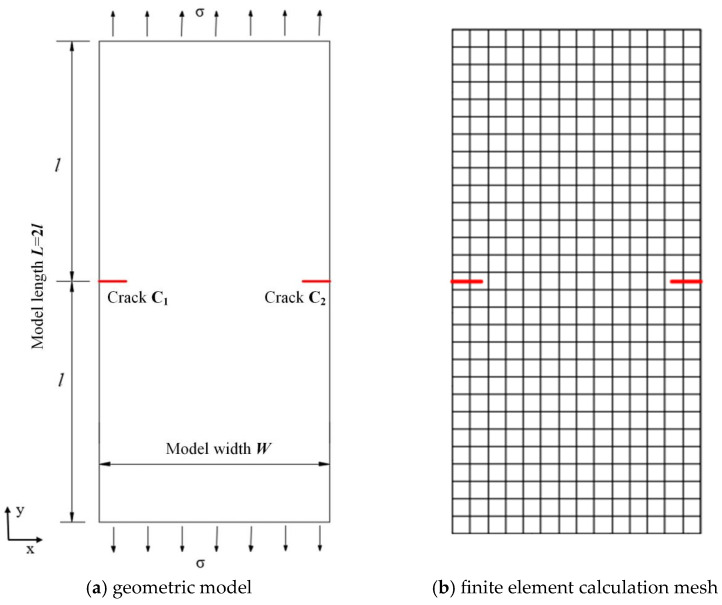
Computational model for double horizontal cracks.

The computational model of the example is shown in Figure 5. The geometric model has a width *W* of 1 m and a length *L* of 2 m. There are two single-edge cracks C_1_ and C_2_ on the left and right sides of the model’s midline, respectively, both with a length of *a*. Uniform tensile stress σ of 1.0 MPa is applied to the upper and lower boundaries of the model. The coordinates of the two end nodes of crack C_1_ are (0,1.0) and (a,1.0), and the coordinates of the two end nodes of crack C_2_ are (2.0−a,1.0) and (2.0,1.0).

Material parameters of the model: Young’s modulus *E* = 2.1 × 10^11^ Pa, Poisson’s ratio *μ* = 0.3. The analytical solutions of the stress intensity factor can be obtained with reference to the *Stress Intensity Factor Handbook*, and the calculation formulas are expressed as

(20) $$K_{I}\;=\;\sigma \sqrt{\pi a}g\left(\xi \right)$$

(21) ξ=2a/W

(22) $$g\left(\xi \right)=(1.122-0.561\xi -0.205\xi^{2}+0.471\xi^{3}-0.19\xi^{4})/{\left(1-\xi \right)}^{0.5}$$

The crack growth angle in the local crack tip coordinate system can be expressed as follows:

(23) $$\theta =2\tan^{-1}\left(\frac{K_{I}-\sqrt{{\left(K_{I}\right)}^{2}+8{\left(K_{II}\right)}^{2}}}{4K_{II}}\right)\;\;\;\;\mathrm{for}\;K_{II}\neq 0$$

(24) $$\;\;\theta =0\;\;\;\mathrm{for}\;K_{II}=0$$

**Figure 6:**
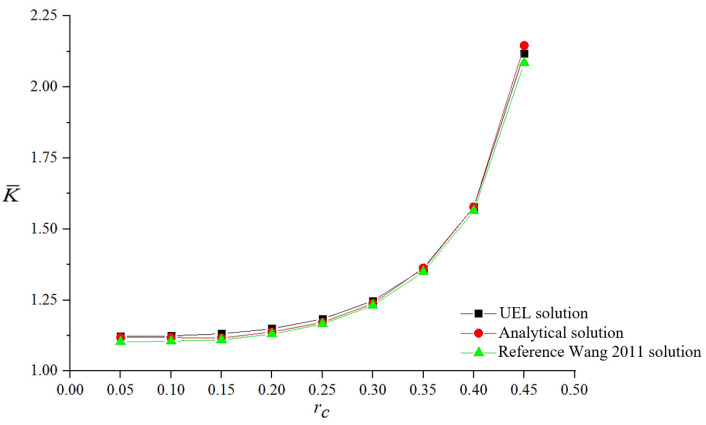
Stress intensity factors under different scale factors.

Define a dimensionless quantity $r_{c}\;=\;a/W$ as the ratio of crack length to model width, and another dimensionless quantity $\;\bar{K}\;=K/(\sigma \sqrt{\pi a})$ (or the symbol you adopt in the text). The user-defined unit program is adopted to conduct static propagation analysis on the model. The stress intensity factor $\;\bar{K}$ under different $r_{c}$, different mesh densities and different integration ratios $r_{a}$ is investigated. As can be seen from Figure 6, the stress intensity factor values solved by the user-defined unit program are basically consistent with the analytical solutions, and the error is controlled within 1%. This indicates that the program compiled by the author can solve the static propagation problem of multiple cracks with relatively high precision, verifying the feasibility of the user-defined unit program.

**Figure 7:**
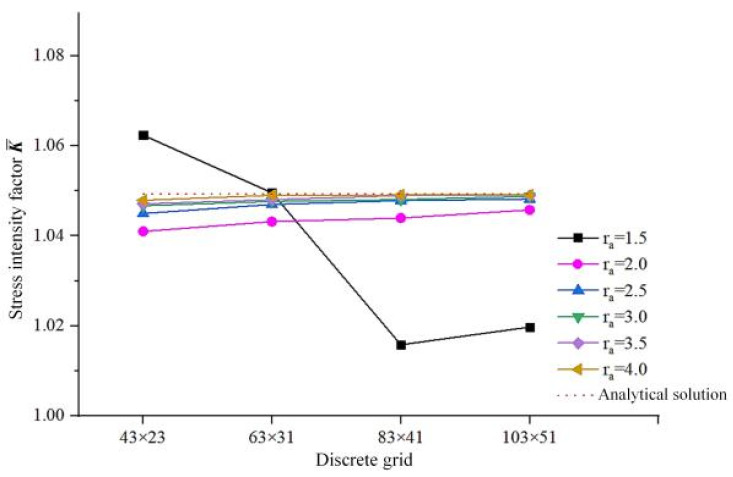
The curve of variation in stress intensity factors.

The following conclusions can be drawn from Figure 7:

(1) The mesh density affects the accuracy of the calculated results. As the mesh refinement increases, the stress intensity factors of K gradually approach the analytical solution, while the calculated results tend to stabilize when the mesh density reaches a certain level.
(2) The J-integral domain factor $r_{a}$ affects the accuracy of the calculated results. When $r_{a}$ < 2.5, the error relative to the analytical solution is relatively large; when $r_{a}$ = 1.55, oscillations appear in the calculated results. When $r_{a}$ > 2.5, the solved stress intensity factor tends to be stable. Therefore, a value of 3.0 can be adopted for the J-integral domain factor K in the static propagation simulation of multiple cracks.

## 5. Numerical Simulation

### 5.1. Numerical Example Simulation of Quasi-Static Crack Propagation in Multi-Crack Systems

The computational model adopted for static crack simulation is used to perform the quasi-static propagation simulation of multiple cracks. The initial crack length a is 0.15 m, the propagation step size is set to 0.04 m, and the mesh density is 23 × 41. Based on the results obtained from the static crack propagation simulation of multiple cracks, the J-integral radius r is taken as 3.0.

**Figure 8:**
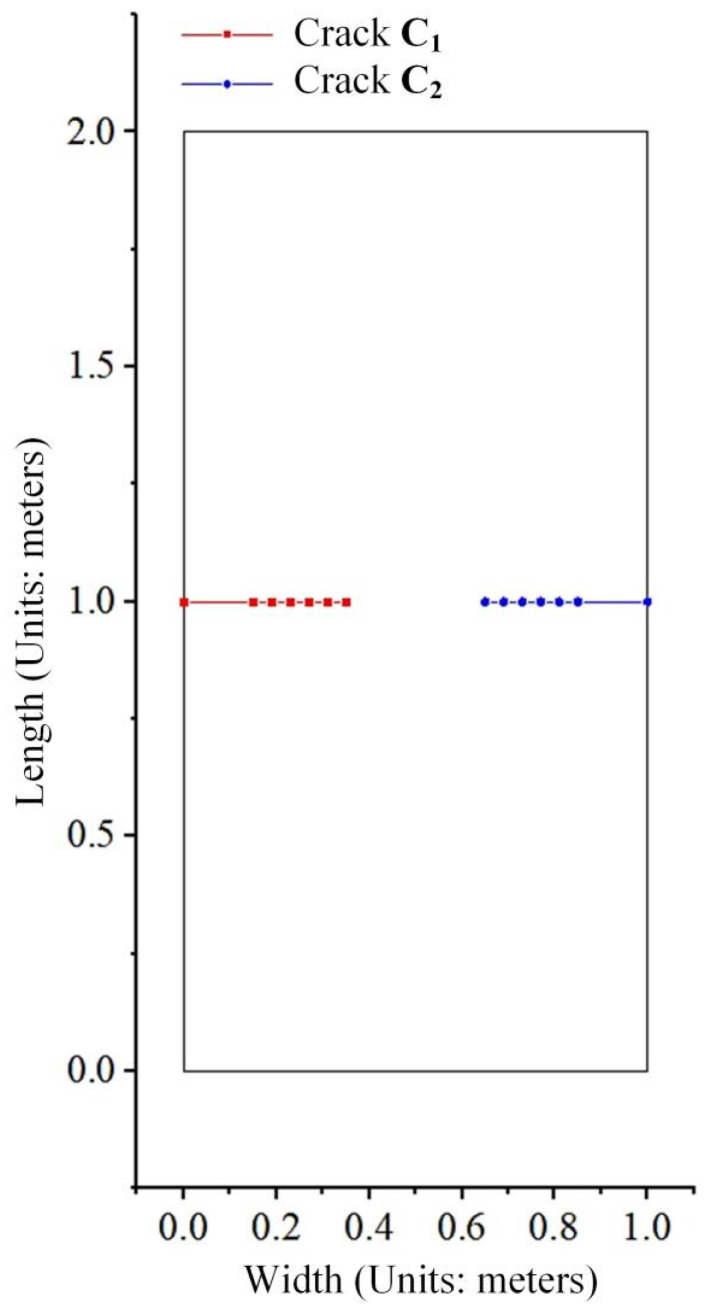
Multi-crack propagation paths.

For the multi-crack propagation problem described in Figure 8, the external load is perpendicular to the crack surfaces, which corresponds to a typical Mode I fracture. According to fracture mechanics theory, the crack propagation direction should be parallel to the original crack orientation. The crack propagation paths depicted in Figure 8 are in good agreement with this rule.

Table 1 lists the stress intensity factor values calculated by the user-defined unit program, the numerical results from the program reported in Reference [20], and the analytical solutions. By comparing the numerical solutions obtained by the proposed program with the analytical solutions, it can be found that the relative error is less than 0.9%, which meets the requirements of computational accuracy. The relative error = |KUEL solution − KAnalytical solution|/|PAnalytical solution|.

### 5.2. Four-Point Bending Test of Concrete Beams

**Figure 9:**
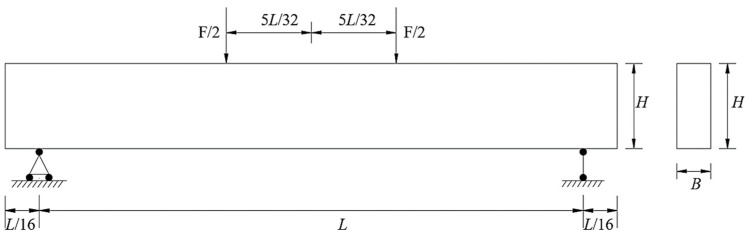
Bending test model of the concrete beam.

The geometric dimensions of the adopted concrete beam are length *L* = 1600 mm, height *H* = 250 mm, and thickness *B* = 100 mm, as illustrated in Figure 9. The material properties of the concrete beam are presented in Table 2.

**Figure 10:**
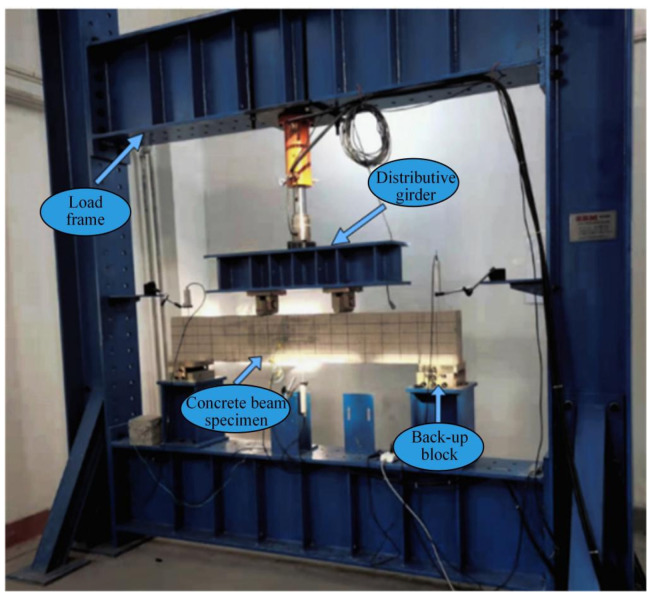
Loading equipment for the concrete beam bending test.

The equipment adopted in the test includes: manual oil pump, jack, distribution beam, loading frame, magnifying glass, and reading microscope. The loading apparatus is shown in Figure 10.

**Figure 11:**
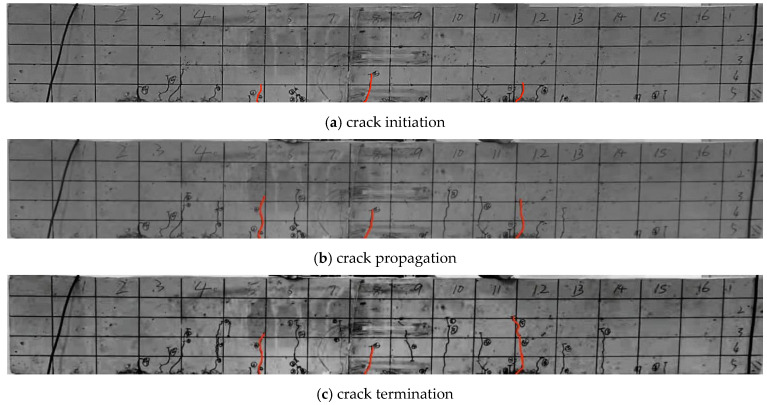
Crack Propagation Paths of the Test.

Bending tests were carried out on the concrete beam specimens with **graded loading** applied until the beam failed. The load value at each stage is listed in Table 3, and the crack propagation path of the concrete beam is depicted in Figure 11.

### 5.3. Numerical Simulation of Multi-Crack Propagation in Concrete Beams

**Figure 12:**
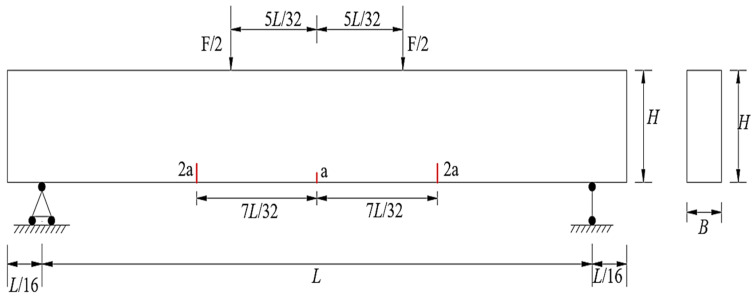
Schematic diagram of initial cracks in the concrete beam.

Crack propagation simulations of the concrete beam shown in Figure 12 were carried out using the ABAQUS-XFEM module and the self-developed program in this paper. The loading scheme is consistent with that given in Table 3. The initial length a of cracks in the pure bending segment is 30 mm, and the initial length 2a of cracks in the non-pure bending segment is 60 mm. The computational model is assumed to be in a plane stress state. According to the simulation results of the example, the mesh near the cracks is refined, and the J-integral radius is set to three times the side length of the crack tip element.

**Figure 13:**
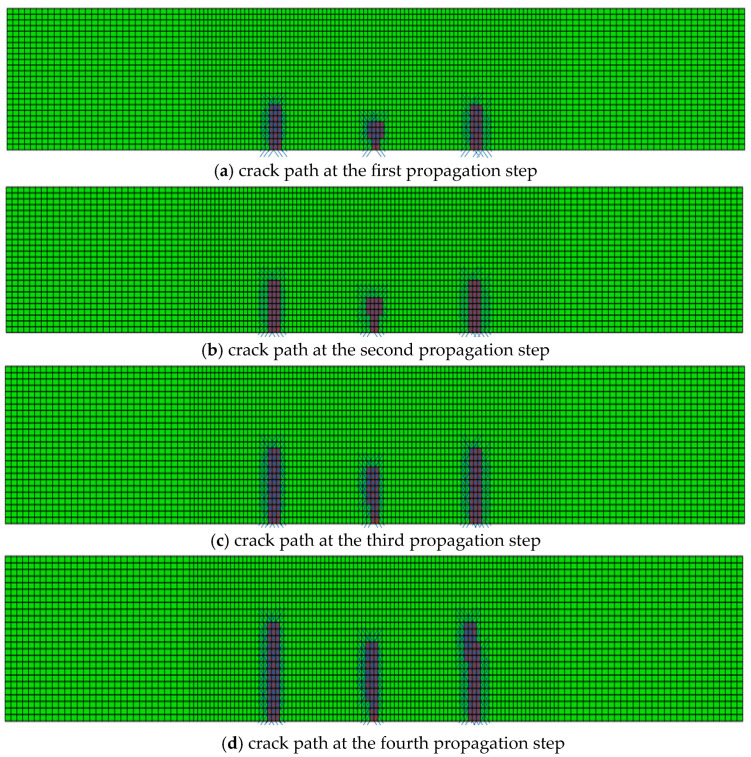
Crack propagation paths obtained by the user-defined element program.

**Figure 14:**
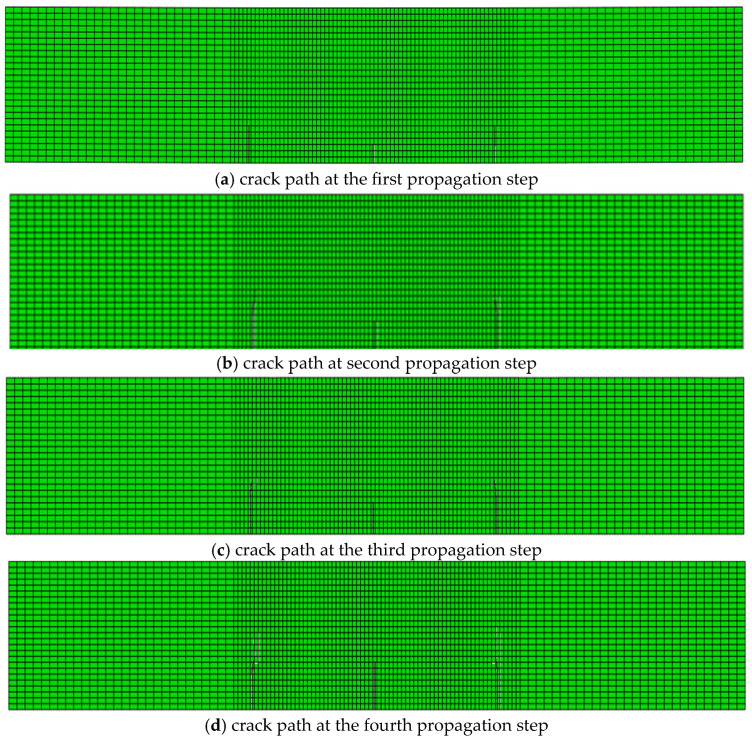
Crack propagation paths solved by the XFEM module.

**Figure 15:**
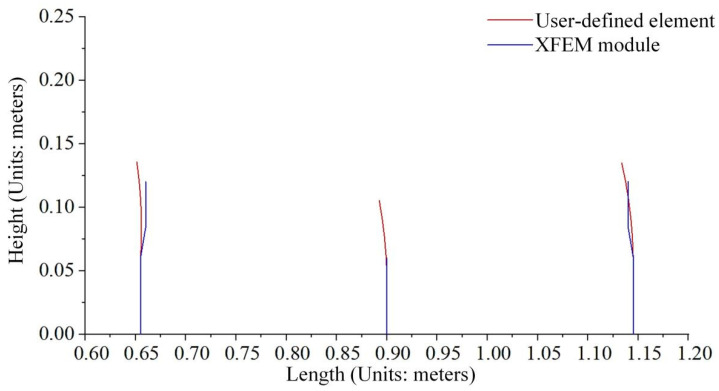
Comparison of the crack propagation paths.

Crack propagation simulation was conducted based on the model depicted in Figure 12. Figure 13 presents the crack propagation path of the concrete beam in four stages, obtained through calculations using a user-defined element program. Figure 14 illustrates the crack propagation path of the concrete beam calculated using the XFEM module in ABAQUS. Figure 15 compares the final crack propagation path obtained through the XFEM module with that obtained using the user-defined element program.

It can be seen from the crack propagation paths in Figure 13, Figure 14 and Figure 15 that cracks in the pure bending segment propagate approximately perpendicular to the bottom surface of the beam, while cracks in the non-pure bending segment first propagate perpendicular to the bottom surface and then gradually extend toward the loading points. The results solved by the self-developed program in this paper are basically consistent with those obtained by the XFEM module.

However, as shown in Figure 14, for the crack propagation paths solved by the XFEM module, the crack tip can only be located on element boundaries and cannot lie inside elements. To achieve higher computational accuracy, only mesh refinement can be adopted. By comparing the numerically simulated crack paths (Figure 15) with the experimentally measured crack propagation paths (Figure 11), it can be observed that the crack propagation trend simulated by XFEM is basically consistent with the experimental results, and the crack propagation paths solved by the proposed program in this paper are in better agreement with the experimental data.

## 6. Conclusions

Based on the basic principles of the extended finite element method (XFEM), an analysis program incorporating crack enrichment elements was compiled using ABAQUS-UEL. Typical multiple cracks were selected for static and quasi-static propagation simulation, and the correctness of the program was verified by comparing the stress intensity factors and crack propagation paths with the analytical solutions. The crack propagation paths were obtained from the four-point bending test of concrete beams, and the cracks in the pure bending section and non-pure bending section were taken as the initial cracks. The ABAQUS-XFEM module and the program developed in this paper were respectively used to simulate and analyze the crack propagation of concrete beams, and the following conclusions are drawn:

(1) When the mesh density reaches a certain level, increasing the mesh density will not significantly reduce the error of calculation results. Only by performing local mesh refinement on the elements near the cracks and adopting relatively sparse meshes for the other parts can high calculation accuracy be achieved with a limited number of meshes, which reduces the calculation cost and improves the calculation efficiency.
(2) The J-integral region factor has an influence on the calculation of stress intensity factors. The error fluctuates greatly when the factor is less than 2.5, while the error fluctuation becomes small when the factor is greater than 2.5.
(3) The error between the simulation results of the program developed in this paper and the theoretical solutions is less than 0.9%, which meets the requirements of calculation accuracy, indicating that the program compiled in this paper can accurately simulate the crack propagation of typical fracture mechanics models with cracks.
(4) The stress distribution solved by the ABAQUS-XFEM module is basically consistent with that obtained by the program developed in this paper in terms of variation trend. Based on the crack propagation paths obtained from the four-point bending test of concrete beams, the crack propagation paths simulated by the program developed in this paper have a higher degree of agreement with the experimental results than those simulated by ABAQUS-XFEM. The results show that the program developed in this paper can effectively predict the crack propagation paths of concrete beams and obtain the multiple crack propagation laws of concrete beams, which can provide a reference for the structural safety design and reinforcement of concrete beams.

## Figures and Tables

**Table 1:** Stress intensity factors at each propagation step.

Propagation Step	1	2	3	4	5	6
$K_{I}$	0.7699	0.8780	0.9875	1.1070	1.2461	1.4177
Reference [20]	0.7681	0.8743	0.9818	1.0975	1.2354	1.4101
Analytical solution	0.7765	0.8842	0.9924	1.1098	1.2480	1.4259
The relative error/%	0.8499	0.7012	0.4937	0.2523	0.1522	0.5751

**Table 2:** Material properties of the concrete beam.

Elastic Modulus/GPa	Poisson’s Ratio	Fracture Energy/N·m^−1^	Tensile Strength/MPa
24.94	0.2	102.2	1.43

**Table 3:** Graded loading.

Load Stage	Load per Stage/kN	Cumulative Load F/kN
0	0	0
1	3.86	7.72
2	2.8	13.32
3 (cracks initiate)	0.965	15.25
4	3.86	22.97
5	3.86	30.69
6	3.955	38.6
7	1.93	42.46
8	1.93	46.32
9	2.14	50.6
10	1.93	54.46
11	2.77	60

## Data Availability

The original contributions presented in this study are included in the article. Further inquiries can be directed to the corresponding author.

## Appendix A

The level set function:
for i=1:N
x=XY(i,2);
y=XY(i,3);
l=sqrt((x1-x0)*2+(y1-y0)*2);
phi=(y1-y0)*x+(x1-x0)*y+(x0*y1-x1*y0);
Vdist=phi/l;
Mdist=([x,y]-XYG(NumPG,:))*t’;
End
The judgment of the element type:
for j=1: NNpe
dg(j)= VdisNG(Top(Ie,j));
dng(j)= MdisNEG(Top(Ie,j),1);
end
dmin= min(dg);
dmax= max(dg);
dminn= min(dng);
dmaxn= max(dng);
if dmin*dmax<=0 & any(dng <= 0)
if dmin*dmax==0
for j=1:NNpe
if dg(j)==0 & gdln(Top(Ie,j),1)==2
gdln(Top(Ie,j),1)= 4;
end
end
else
for j=1:NNpe
if gdln(Top(Ie,j),1)==2
gdln(Top(Ie,j),1)= 4;
end
end
end
end
if dmin*dmax<=0 & dminn*dmaxn<=0
for j=1:NNpe
gdln(Top(Ie,j),1)=10;
end
end
The setting of crack-tip function control region:
mm=3;
dd=0.3;
RR=mm*dd;
toll=1e-8;
if max(abs(dmax),abs(dmin))<RR+toll & max(abs(dmaxn),abs(dminn))<RR+toll
for j=1:NNpe
gdln(Top(Ie,j),1)=10;
end
end
rc=1.2;
toll=1e-8;
if any(abs(VdisNG(Top(Ie,:)))<=rc & abs(MdisNEG(Top(Ie,:))<=rc))
for j=1:NNpe
gdln(Top(Ie,j),1)=10;
end
end
The crack-tip enrichment function:
rt= (X-XYG0(1))*tx + (Y-XYG0(2))*ty;
rn= -(X-XYG0(1))*ty + (Y-XYG0(2))*tx;
r2= (X-XYG0(1))**2 + (Y-XYG0(2))**2;
r= sqrt(r2);
rr= sqrt(r);
theta= atan2(rn,rt);
F(1)= rr*sin(theta/2);
F(2)= rr*cos(theta/2);
F(3)= rr*sin(theta/2)*sin(theta);
F(4)= rr*cos(theta/2)*sin(theta);
The calculation of stress intensity factors:
KI=ijvalorI*simetria*Eprima/2.0d0;
KII=ijvalorII*simetria*Eprima/2.0d;
ijinfer=(KI**2/Eprima + KII**2/Eprima);
porcen=ijinfer/ij/simetria*100;
KIIinfer=dsqrt(ij*simetria*Eprima-KI**2.0d0);
angMCS=dabs(dacos((3.0d0*KII**2+dsqrt(KI**4+8.0d0*
KI**2*KII**2))/(KI**2+9.0d0*KII**2)));
if (KII.gt.0) angMCS=angMCS*-1.0d0
angMCS=angMCS*180.0d0/dacos(-1.0d0);
return
END
